# The design, development, and evaluation of a prototypic, prosthetic venous valve

**DOI:** 10.1186/1475-925X-7-25

**Published:** 2008-09-19

**Authors:** Matt T Oberdier, Stanley E Rittgers

**Affiliations:** 1 Department of Biomedical Engineering, The University of Akron, Akron, OH 44325, USA

## Abstract

**Background:**

Chronic venous insufficiency is a serious disease for which there is no clearly successful surgical treatment. Availability of a proven prosthetic vein valve could provide such an option by reducing venous reflux while permitting normal antegrade flow.

**Methods:**

A new prosthetic vein valve design has been developed which mimics the function of a natural valve by ensuring complete closure of the leaflets with minimal obstruction for antegrade flow. A 2:1 mock-up of the device was tested to evaluate its ability to prevent regurgitation and several key modifications were made. A subsequently re-designed 1:1 prototype was then built in 4 slightly different size configurations and then each tested under physiologic conditions of pulsatile flow in both supine and standing positions.

**Results:**

Each of the configurations showed acceptable amounts of antegrade resistance and effective orifice area and showed low values of regurgitation and % reflux with two of the prototype configurations (flange lengths of 2.5 mm and 3.75 mm) having corresponding values of <2.5 mmHg-min/L, >97%, 11 mL, and 36%, respectively. These values are particularly striking when compared to the corresponding regurgitation and % reflux values of 60 mL and 205%, respectively, when no device is present.

**Conclusion:**

The results of this study show that this prototype vein valve design is capable of providing significant relief of reflux under realistic conditions without inducing any increase in antegrade flow resistance and warrants further testing with *in vivo *models.

## Background

In the United States, chronic venous insufficiency (CVI) afflicts ten to thirty-five percent of the adult population to some extent [1] and is the seventh most common chronic debilitating disease [2]. Ulceration associated with CVI occurs at a rate of up to one and a half percent and in four percent of patients over sixty-five years of age in industrialized populations [2]. CVI is distinguished from other chronic venous disorders by a variety of alterations of the skin due to prolonged venous hypertension [3]. Incompetent venous valves directly result in reflux, which in turn produces hypertension and causes the most serious symptoms of CVI including ulcers [4]. Increasing venous reflux and decreasing calf muscle pump ejection fraction have been shown via air plethysmography to be related to the incidence of ulceration [5].

Current CVI management strategies include external compression devices, intermittent pneumatic compression, other adjuvants (including dressings and skin substitutes), and drugs while surgical alternatives include but are not limited to ligation and stripping, thermal ablation, sclerotherapy, valvuloplasty, and venous segment transfer [6]. However, non-operative therapies remain the primary treatment for CVI and ulceration [7]. Unfortunately, non-operative techniques are only palliative in that they make the symptoms of CVI tolerable while not treating the underlying cause of the disease (i.e. venous reflux and hypertension).

It is believed that a prosthetic venous valve is capable of restoring normal hemodynamics as an alternative to surgery or for those patients without surgical options. Currently, there are no commercially available prosthetic venous valves as previous efforts have been hampered by late failure and biocompatibility issues [8]. To date, only two independently designed bioprosthetic valves have undergone clinical trials [9-12] although several others have been evaluated *in vitro *and in animal models.

Wang et al. [13] were among the first to create and test bioprosthetic vein valves when they mounted bovine jugular valves in straight, tapered, and curved conduits. The devices were evaluated in an *in vitro *system where it was determined that different pulse pressure thresholds were necessary to "reestablish the CO [closure opening] operation mode" for each conduit geometry. Similarly, Delaria et al. [14] evaluated fresh and glutaraldehyde fixed bovine jugular vein conduits in an *in vitro *flow system and determined that the performance of the two valves is similar while fixation theoretically renders biocompatible and nonthrombogenic tissue. Therefore, it was suggested that when mounted and stented, a glutaraldehyde fixed valve has the potential to be the first clinical solution to CVI.

The most extensive work was performed by Pavcnik et al. [15-21] who iterated on a bioprosthetic venous valve consisting of a small intestinal submucosa (SIS) leaflet material sutured to an expandable square stainless-steel wire stent. The device featured sinuses and barbs for vascular wall anchoring and was designed to be deployable via catheter. Wire thickness determined valve size and expansile force where ideal geometry was determined by constraining the diagonal axis of the square stent to a length of πr based on a vessel circumference of 2πr [15]. When implanted in an ovine external jugular vein model, the valve restored antegrade circulation with little or no reflux and the SIS leaflet was shown to undergo endothelialization, neovascularization, and incorporation into the native vessel wall on the order of several months [18]. However, in several animals, tilting of the valves lead to moderate reflux and thrombosis [18] prompting a second-generation design that consisted of either a stainless-steel stent attached to the apex of the original or a nonrestrained nitinol double-stent [19]. The second-generation design effectively corrected for valve misalignment; nonetheless, sizing with the nitinol version and intimal hyperplasia with the stainless-steel device caused dysfunction [19]. A later study indicated that spatial orientation is important and should correspond to that of the native valve [20]. Although the previously described efforts by Pavcnik et al. [21] have shown favorable *in vivo *results in the short-term, a human feasibility study revealed that occlusion occurred in four of fifteen valves at 12-month follow-up. Additionally, three oversized valves opened too much resulting in leaflet-to-wall attachment and four devices exhibited undesirable leaflet pliability from adverse healing. Hence, the potential for clinical application of this bioprosthetic venous valve remains uncertain.

Work on synthetic valves has been more limited than those of bioprosthetic design. A synthetic bileaflet valve made of platinum or pyrolyte carbon covered titanium was reported by Taheri and Schultz [22]. In nine canines, the device restored retrograde flow and remained patent in the short-term; however, within the initial three months, two of the valves migrated or were misoriented leading to failure. Within two years, all canines developed symptoms of CVI due to intimal hyperplasia. Sathe et al. [23] also created a synthetic venous valve, which is composed of a clinically approved polymer.

The objectives of the subsequently described prosthetic design were to preserve natural anatomic venous valve features while improving upon a conceptual first-generation device described in a prior patent [24] for which no quantitative testing was performed. The novel second-generation design features are also presented in a patent application currently under review [25].

## Methods

**Figure 1:**
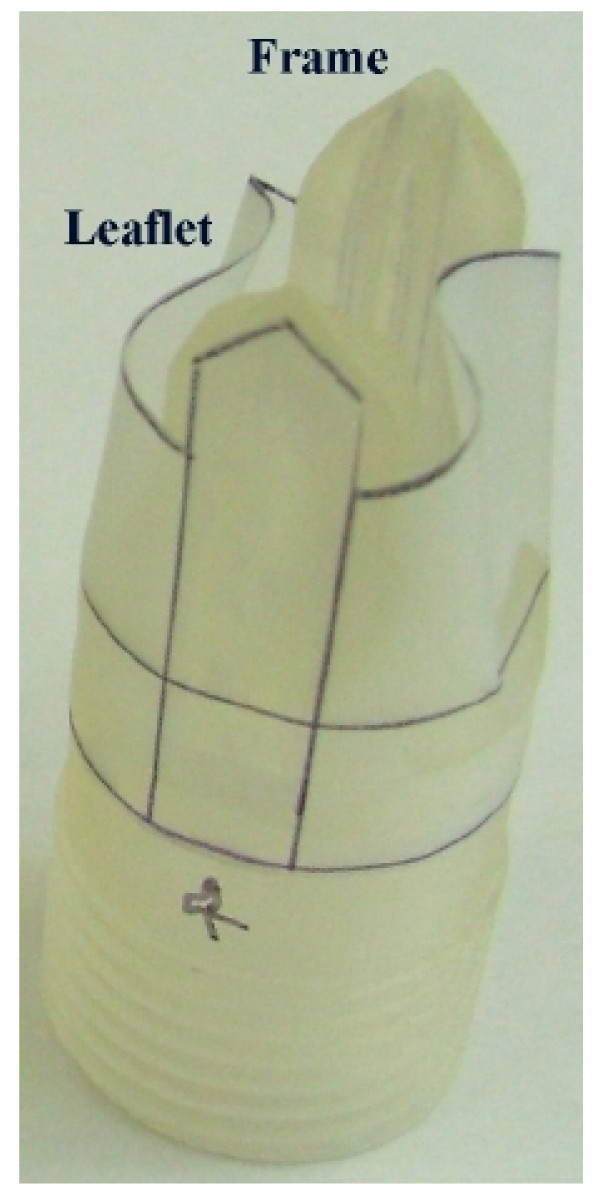
**The two main components of the novel, second-generation design: a solid frame and a flexible leaflet.** The threaded adaptor extension used to improve alignment and prevent leakage during *in vitro *testing is shown distal to the lower line. (2× scale model).

From the natural anatomy, the most advanced second-generation valve maintained sinus regions behind each of two leaflets, which were in a normally open position. Preserved features from the first-generation design include the integration of two distinct entities: a solid frame and a flexible leaflet (Figure 1). More specifically, the frame consists of a circular base from which two opposing struts project proximally along the valve axis and where the flexible leaflet is anchored. Novel features of the second-generation valve include the addition of inward directed flanges to allow for precise matching of leaflet material to closure path, a proximal leaflet contour to create a single transverse plane of closure, shoulders on either side of the struts for frame reinforcement and leaflet support, and a beveled leaflet attachment surface that allows for the creation of sinuses. Figure 1 defines the solid frame and flexible leaflet for the second-generation design.

Frame design began by rendering the valve flange in two-dimensions (in the transverse plane) followed by three-dimensional axial contouring. Then, the frame geometry was used to mathematically define a two-dimensional leaflet template. Next, the frame and leaflet were fabricated at a super-anatomic scale (2×) and a complete valve created by wrapping the leaflet template around the frame. The valve was then evaluated in a mock-up *in vitro *flow system (2:1 dynamic scaling) and, based on performance, the need for two additional frame features was defined: strut shoulders and a beveled leaflet attachment surface. Finally, a matrix of devices from the most advanced design were fabricated at anatomic scale (1×) and evaluated in a physiologically-based, *in vitro *flow system (1:1 dynamic scaling).

### a. Two-dimensional frame design in the transverse plane

Inward directed flanges were introduced to occupy space in the transverse plane such that the frame base circumference exactly matches the leaflet closure path ensuring that pleating or gapping does not occur upon leaflet closure. One flange geometry fit this criterion while providing gradual curves and a simple mathematical relationship from which to design. In this configuration, each flange consists of two half-circles connected by two inverted quarter-circles of the same radius, which converge to a point directed towards the valve lumen. At the points where the flange intersects the frame base circumference, the half circles are connected with a straight line producing a flat flange "back". The point at which the flange back and half circle met was constrained as tangent to the circular base.

A design equation was defined in which the frame base circumference and the closure path were balanced. Utilization of symmetry in the quarter-plane resulted in:

(1) $$\frac{\pi R}{2}=\frac{3\pi r}{2}+r+Q$$

where R = the valve radius,

r = the flange radius, and

Q = a constant that compensates for the flat flange back and tangent point.

**Figure 2:**
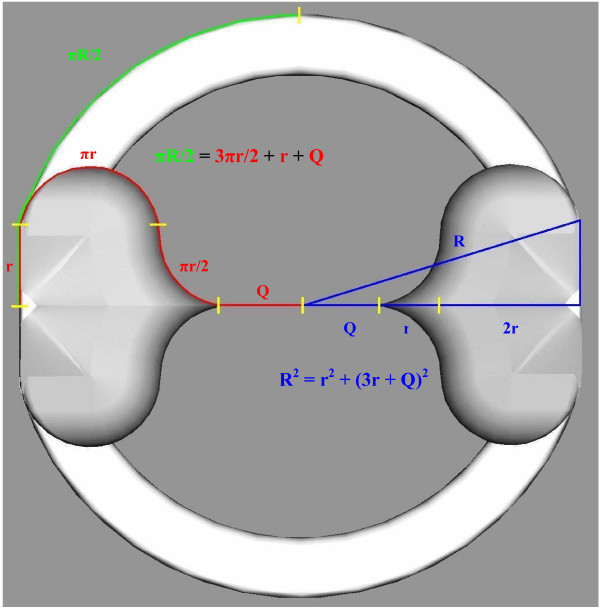
**Geometric relationships and frame design equations in the transverse plane.** Equation 1 is represented on the left where it is the goal to match the valve circumference (green) to the closure path (red). Equation 2 is represented on the right where the objective is to compensate for the flat flange back and tangent point. When solved simultaneously, the system provides a solution for both the flange radius, r, and Q where the valve radius, R, is the independent parameter.

When Q equals r, the distance along a line from the central valve lumen and perpendicular to the flat flange back (subsequently labeled the y-axis) is exclusively dependent on the flange geometry and independent of the valve radius, R. Hence, Q cannot equal r and solution requires a second design equation that is obtained by applying the Pythagorean Theorem to the variables already defining the frame. The second design equation is:

(2) *R*^2 ^= *r*^2 ^+ (3*r *+ *Q*)^2^

(Figure 2 depicts the frame geometry and design relationships in the transverse plane.) Choosing a valve radius of R = 6.25 mm based on observations from a normal volunteer study performed by our group [26] allowed Equations 1 and 2 to be solved simultaneously. For R = 6.25 mm, it was found that r = 1.37 mm and Q = 1.98 mm.

A minor limitation of the frame design is that the base circumference side of Equation 1 does not take the flat flange back into account. Nonetheless, this assumption results in an error of approximately 1E-5 mm for the quarter-plane closure path, which is not considered significant.

### b. Three-dimensional frame design

With design in the transverse plane finalized, the flange geometry was then extended into a three-dimensional object with the goal of optimizing hemodynamics and biocompatibility. More specifically, zones of stasis and excessive local shear stress were intended to be diminished along the flange such that thrombus formation and blood damage would be minimized. Additionally, it was recognized that flange contouring has the potential to limit pressure drop and thus, minimize resistance. Therefore, the flanges were extruded with minimal slope discontinuities in the axial direction and tapered at their proximal and distal ends.

### c. Leaflet design

Based on the second generation frame, leaflet design considerations were defined in anticipation of future *in vivo *application. For example, it was desirable to minimize material surface area to limit thrombogenicity. Further, it was important to decrease leaflet flexion angle because the proximal base edge is a high stress region and thus, would be the first area to fatigue from cyclic loading. Unfortunately, these criteria work opposite to one another in that decreasing the angle of leaflet closure (i.e. decreasing cyclic stress) requires axial lengthening of the flange and therefore, increasing surface area. Given the aforementioned considerations, it was useful to create a design constraint whereby all points of the proximal leaflet edge are required to coapt in a single transverse plane. Such a closure plane was defined to be at the midpoint of the flange and allowed the frame and leaflet surface areas to be minimized for a specific flange length.

**Figure 3:**
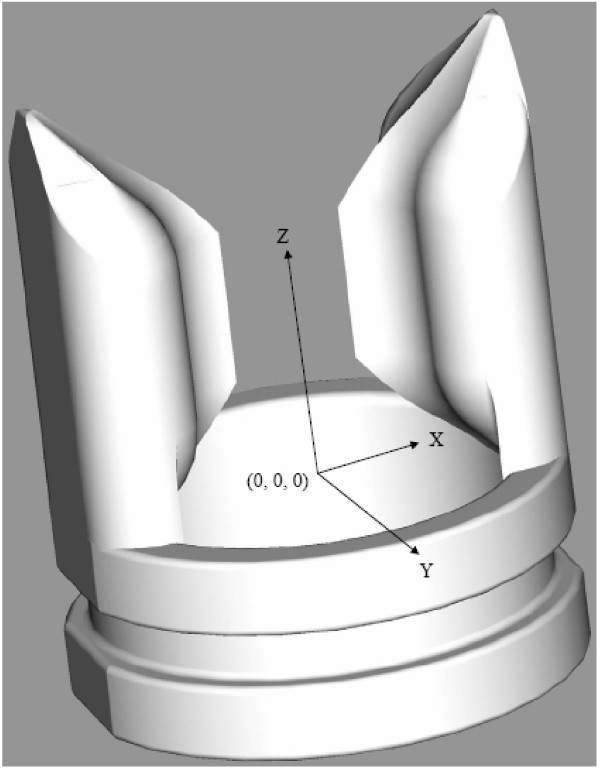
The three-dimensional Cartesian coordinate system utilized for leaflet design.

However, the first step in implementing the desired design feature was to define a three-dimensional Cartesian coordinate system based upon the optimized frame design. The establishment of such a coordinate system allows all points of the frame base circumference and closure path to be defined relative to one another in three-dimensions. The reference planes were logically assigned relative to the frame geometry. More specifically, the xy-plane is parallel to the transverse plane where the x-axis is directed towards the flange back and the y-axis towards the valve sinus. The z-axis is perpendicular to the xy-plane and follows the axis of the valve lumen. The point (0,0,0) is defined to be on the valve axis in the xy-plane and at the proximal base edge in the yz- or xz-planes. Figure 3 shows the three-dimensional coordinate system with respect to the second-generation frame.

**Figure 4:**
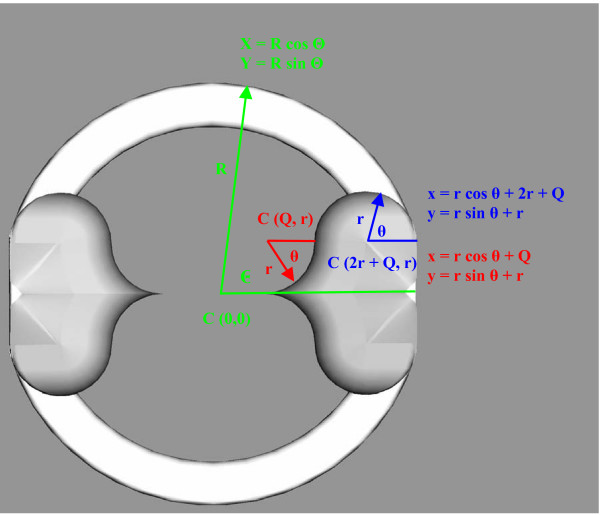
Cartesian definitions for point mapping and leaflet design in the transverse plane.

Next, the frame was viewed in the xy-plane and points from the radial edge of the base were mapped to their corresponding points on the flange during full closure using a spreadsheet program (Microsoft Excel, Microsoft Corporation, Redmond, WA). In two-dimensions, the distance between these points is the minimum length the leaflet needs to have to guarantee full closure. For the mathematical details of the point mapping, see the Appendix.

Finally, the minimum leaflet length that guarantees full closure was calculated as the three-dimensional resultant of the point on the radial base edge to its corresponding point in the closure plane. This was done using the previously described three-dimensional mapping techniques and the following relationship:

(10) $$L=\sqrt{{\left(X-x\right)}^{2}+{\left(Y-y\right)}^{2}+Z-z{)}^{2}}$$

where L = the minimum length of the leaflet for full closure

The valve base height was added to L and provided an overlap between frame and leaflet for the application of adhesive.

**Figure 5:**
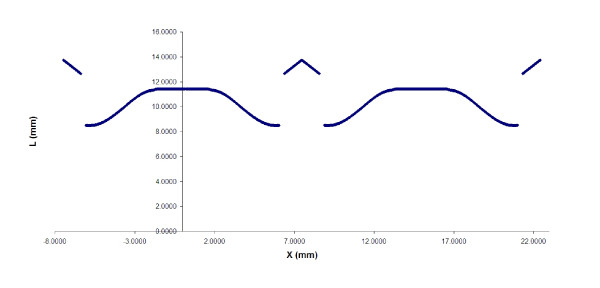
The 2-D leaflet template with L plotted versus X by the spreadsheet program.

The tabulation of corresponding values of X and L provides a series of data points that create one-quarter of a two-dimensional leaflet template. When symmetry is utilized and additions for the flange back are made, a complete template is produced. The template creates a leaflet when wrapped around the frame. Figure 5 is the leaflet template as plotted by the spreadsheet program.

### d. Fabrication

The completed valve prototype consists of a combination of the solid frame and flexible leaflet. The frame was accurately and precisely modeled using computer-aided design (CAD) software (ProE 2000 i^2^, Product Development Company, Needham, MA) and then fabricated with stereolithography (SLA) rapid prototyping technology that utilized a photo-activated polymer resin (RenShape^® ^SL 5210, Huntsman Advanced Materials, Salt Lake City, UT) consisting of bis-(epoxycyclohexyl)-methylcarboxylate and 2,2-dimethoxy-2-phenylacetophenone. Upon polymerization, residual resin was removed by submersion in iso-propanol and further cured in a UV oven. The leaflets, on the other hand, were created using a hand fabrication technique. The data points composing the leaflet template were transferred to a different program (MATLAB 6.5, The MathWorks Incorporated, Natick, MA) for 2D plotting. The axes were scaled equally for proportionality and then printed on an 8.5 × 11.0 inch sheet of paper. A copy machine (Canon NP6545, Canon Incorporated, Japan) was then used to reduce the template to a size that corresponded to the valve frame. This size was determined via trial and error and the best fit selected based on circumference. For the mock-up valves (2×), the scaling factor was found to be 54% whereas the prototypes (1×) required a reduction of 50% followed by a subsequent scaling of 57%. Once the proper size template was acquired, the leaflet material was taped over the template and the geometry of the leaflet traced on the material using a black pen. For all created valves (both 2× mock-ups and 1× prototypes), 5 mil (0.1270 mm) thick sheets of BioSpan^® ^(BioSpan^®^, Polymer Technology Group, Berkeley, CA) were used as the leaflet material upon which reference lines for the flange back and proximal base edge were also drawn. The leaflet was then cut out using a surgical scalpel and super-glue was applied to the portions of the leaflet that were to meet on the flange back and below the proximal base edge. Finally, the leaflet was attached to the frame by approximating the center of the leaflet to the flange back and then rolling the valve in either direction to evenly wrap the remainder of the leaflet around the frame base.

### e. 2:1 mock-up, *in vitro *flow system and design modifications

**Figure 6:**
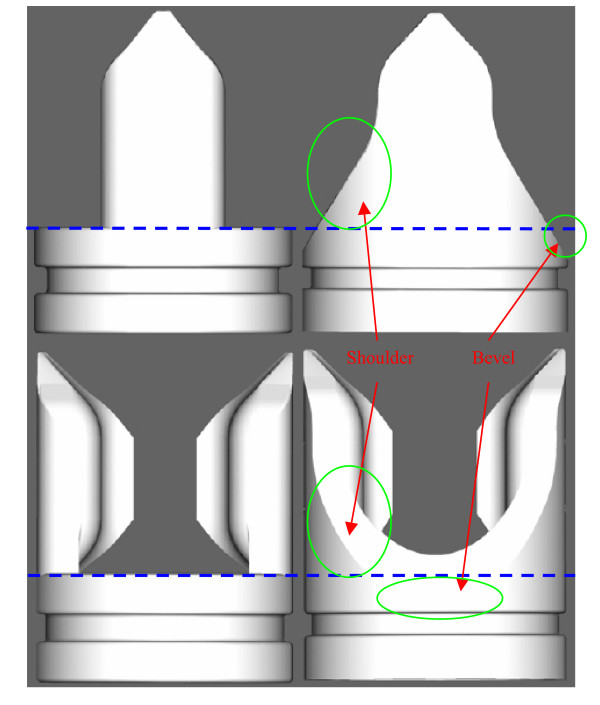
**The original (left) and most advanced (right) second-generation frames as depicted in the yz- (top) and xz- (bottom) planes. **The additional bevel and shoulder features of the most advanced design are labeled and indicated with arrows.

After completing the initial design, the valve was fabricated at 2× scale and inserted into an *in vitro *flow loop featuring a vertical column, steady flow system with water as the test fluid as previously described by our group [26]. Dynamic similarity was maintained from the 1× scale with the goal of studying leaflet-frame interaction under a uniform reversing pressure. Three observations were made that warranted further development of the second-generation design: 1) the leaflets only closed under a physiologically analogous reversing pressure gradient when they were artificially drawn off the tube wall, 2) an undesirable sagging of the closed leaflets was observed in the yz-plane, and 3) the frame was susceptible to breaking where the flanges intersect the proximal base edge when subjected to a bending stress. The first issue was addressed by creating a bevel on the proximal base edge from which the leaflets were adhered that also produced a sinus behind each leaflet. The other two problems were resolved with the addition of a supporting "shoulder" that intersects the flange at the closure plane (when viewed in the yz-plane). In the xy-plane, the shoulder followed the proximal base edge, but did not protrude into the valve lumen. The proximal edge of this shoulder was anticipated to support the leaflet during closure while the remainder of the structure reinforces the flange. Correspondingly, the leaflet design spreadsheet was changed to accommodate the new geometries. The evolution of the bevel and shoulder features is shown relative to the original second-generation frame design in Figure 6.

### f. Design matrix

The aforementioned methodology addresses the issues of conserving leaflet and flange material by defining a single closure plane; however, varying the angle of leaflet flexion and sinus volume was not addressed. Nonetheless, it was recognized that the generic design could be dimensionally modified to accommodate such considerations. A single feature of the flange was identified that influenced both leaflet flexion angle and sinus size and was referred to as the flange length (FL). Physically, the FL was described as the distance in the z-direction between the tapered tips of the fully developed flange. Four different flange lengths were chosen: 1.25, 2.50, 3.75, and 5.00 mm, which had the effect of varying the leaflet flexion angle in a linear range from 38.7 to 11.3°, respectively.

### g. 1:1 physiologically-based, in vitro flow system

After the addition of the bevel and shoulder features, the prototypes were fabricated at 1× scale with threading at the distal end to allow reproducible insertion into the physiologic *in vitro *flow model as described by our group [26]. A blood analog test fluid consisting of 3.45% dextran (Sigma-Aldrich Corporation, St. Louis, MO) in distilled water was utilized where the viscosity was 3.5 cP. The testing protocol and parameters of interest were preserved from the prior study although a less detailed review is concurrently presented.

The *in vitro *flow model was designed to simulate any combination of two body positions (supine and standing) and two dynamic conditions (breathing and ankle flexion). Therefore, a total of four simulations are possible: supine breathing (SUBR), standing breathing (STBR), supine ankle flexion (SUAF), and standing ankle flexion (STAF). Body position was controlled via hydraulic communication between hydrostatic pressure towers and the test section while dynamic condition was determined via the frequency of flow pulsatility provided by a roller pump. A "No Device" case was run in an attempt to simulate the dynamic conditions of CVI in which the valve system is compromised. As the nomenclature indicates, the "No Device" case consisted of executing the test protocol without a device in place where a completely open orifice existed and the system was sealed around the threaded adaptor. The "No Device" case was used to calculate the experimentally determined Effective Orifice Area (EOA) coefficient, C_d_, appropriate for these conditions where the open orifice area was directly measured and Q_rms _and ΔP were obtained from sampled data [26]. C_d _values of 2.32 ± 0.030 and 6.38 ± 0.142 cm.mmHg^1/2^.s^-1 ^were obtained for the SUBR and SUAF simulations, respectively.

**Figure 7:**
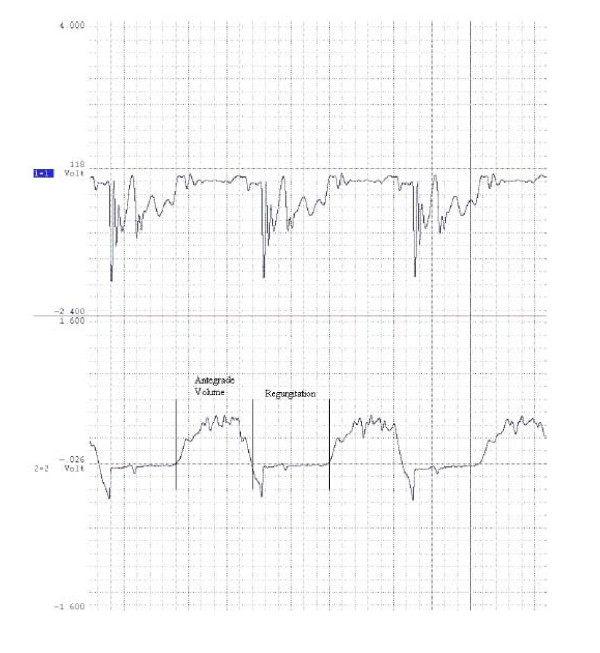
**Representative differential pressure (top) and flow rate (bottom) waveforms from the physiologic system under the STBR condition utilizing a second-generation prototypic valve.** The division of one cycle associated with the calculation of antegrade and regurgitant volumes is shown.

Sampled data included differential pressure and flow rate waveforms (Figure 7) from which Resistance, EOA, Regurgitation, Percent Reflux, Reflux Energy Loss (REL), Antegrade Energy (AE), and Energy Retention parameters were calculated as defined in our prior manuscript [26]. Beyond the parameter definitions that are standard for cardiac valves (Resistance, EOA, and Regurgitation), Percent Reflux, REL, AE, and Energy Retention were developed specifically for the evaluation of venous valves. Briefly, Percent Reflux is the ratio of retrograde to antegrade volume, REL is the energy necessary to re-pump the reflux volume through the valve, AE quantifies the energy produced by the pump to propel the fluid proximally, and Energy Retention compares REL to AE. All values are based on five cycle periods and are presented as means ± standard deviation.

### h. Statistical methods

Since there currently exists no clinically approved '*de facto' *standard device for comparison, it was hypothesized that the second-generation prototypes would significantly improve systemic level hemodynamics (as characterized by the Regurgitation, Percent Reflux, REL, AE, and Energy Retention parameters) when compared to the CVI simulated "No Device" case. Additionally, it was theorized that differing FLs lead to unique *in vitro *dynamic performance for each parameter. Statistical analysis of design matrix data consisted of a one-way analysis of variance (ANOVA) followed by *post hoc *SNK or Dunnet T3 procedures for equal and unequal variances, respectively. A comparison of duplicate prototypes of the same design utilized a series of independent-samples t-tests. Both statistical analyses were performed using a commercially available software package (SPSS 14, SPSS Incorporated, Chicago, IL) with a significance level of 0.05.

## Results

### a. 2:1 mock-up, *in vitro *flow system

Although data was collected as part of mock-up testing, those results are not presented because the developmental value of the simulation was primarily qualitative. However, as previously described, mock-up testing resulted in the addition of the shoulder and bevel features, which provided leaflet support and created sinuses, respectively. It was found that sinuses are crucial for valve actuation upon venous diastole (a retrograde pressure gradient).

### b. 1:1 physiologically-based, *in vitro *flow system

**Figure 8:**
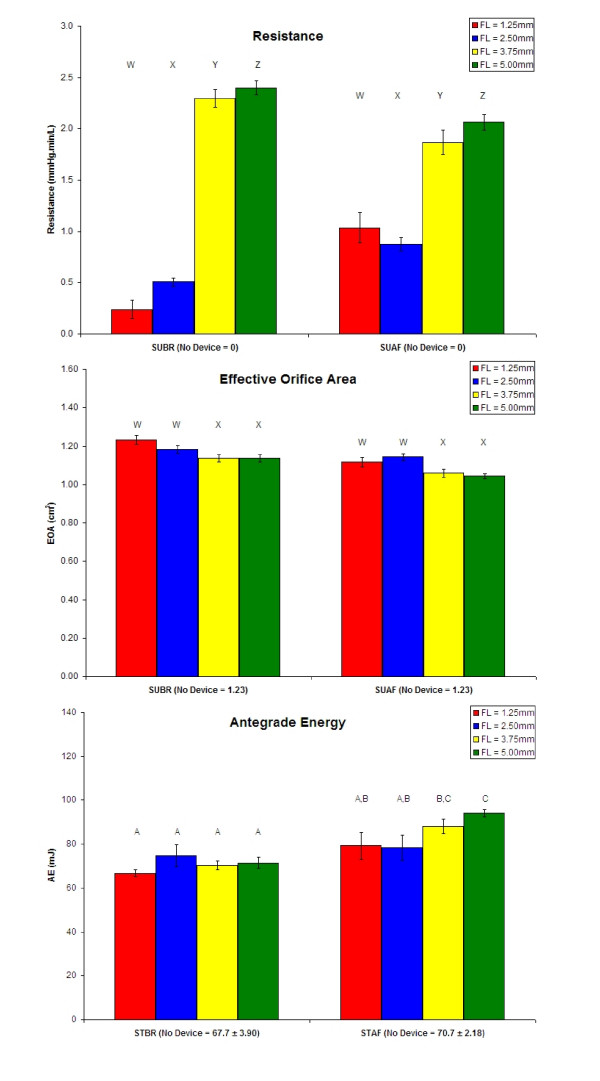
**Means, standard deviations, and statistical groupings for Resistance, EOA, and AE for the FL 1.25–5.00 mm prototypes.** Corresponding values for the "No Device" case for each simulation are presented as part of the x-axis label. Alphabetical labels denote groups that are statistically different from each other (p < 0.05). By convention, the W-X-Y-Z grouping nomenclature (for Resistance and EOA) does not include the "No Device" case as part of the ANOVA whereas the "No Device" case is included in the ANOVA as statistical group A where A-B-C-D nomenclature is utilized (for AE).

Data characterizing antegrade hemodynamic device performance is presented in Figure 8. For the supine position simulations, the devices with FLs of 1.25 and 2.50 mm statistically had the lowest Resistance with both achieving around 0.50 and 1.0 mmHg.min/L for the SUBR and SUAF simulations, respectively. With regard to EOA, devices with FLs of 1.25 and 2.50 mm exhibited statistically superior EOA performance with approximately 1.2 cm^2 ^for both simulations. The "No Device" Resistance was defined as 0 mmHg.min/L whereas the "No Device" EOA was calculated as the cross-sectional area of the open tube. Hence, the "No Device" Resistance and EOA were not included in the ANOVA and the corresponding grouping nomenclature is different from that of the other parameters.

**Figure 9:**
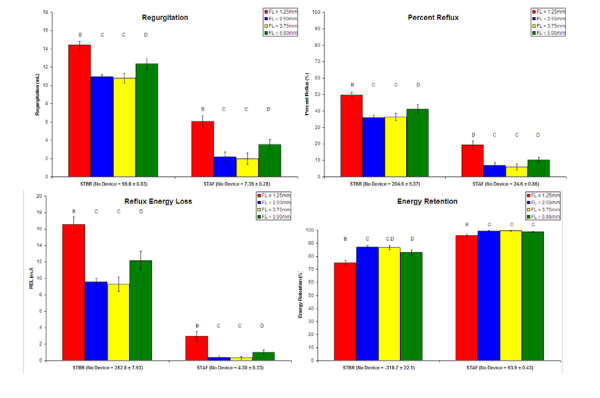
**Means, standard deviations, and statistical groupings for Regurgitation, Percent Reflux, Reflux Energy Loss, and Energy Retention (parameters characterizing retrograde device performance) for the FL 1.25–5.00 mm prototypes.** Values for the "No Device" case for each simulation are presented as part of the x-axis label. Alphabetical labels denote groups that are statistically different from each other (p < 0.05). By convention, the "No Device" case belongs to statistical group A.

Data characterizing retrograde hemodynamic device performance is presented in Figure 9. With regard to the standing position simulations, all devices statistically improved retrograde hemodynamics relative to the "No Device" case. More specifically, the FL 2.50 and 3.75 mm valves statistically outperformed the other two devices in the standing position simulations for the Regurgitation, Percent Reflux, and Reflux Energy Loss parameters. Also, the FL 2.50 and 3.75 mm devices demonstrated favorable performance for the Energy Retention parameter in the STBR simulation. Further, the Antegrade Energy data for the STBR simulation all belong to the same statistical grouping, which includes the "No Device" case. For the STBR simulation, the FL 2.50 and 3.75 mm prototypes demonstrated Regurgitation, Percent Reflux, Reflux Energy Loss, and Energy Retention values around 11 mL, 36%, 9 mJ, and 87%, respectively, as compared to 60 mL, 205%, 283 mJ, and -319% for the "No Device" case, respectively. Data from the STAF simulation were approximately 2 mL, 6%, 0.5 mJ, and 99%, respectively, for the FL 2.50 and 3.75 mm devices and 7 mL, 25%, 4 mJ, and 94%, respectively, for the "No Device" case.

As shown in Tables 1A and 1B for duplicates of the FL 2.50 mm design, intra-design variability in the supine position simulations is statistically significant for all parameters with the exception of SUAF EOA. For the standing position simulations, intra-design variability is statistically significant for all parameters. Multiple prototypes of the FL 5.00 mm design were also created, which show significant differences only for the SUBR Resistance and STAF REL parameters (data not presented).

**Table 1:** 

**A Antegrade intra-design variability as indicated by evluation of duplicate prototypes from the FL 2.50 mm design**.						
	**SUBR**		**STBR**	**SUAF**		**STAF**

Device	Resistance (mmHg·min/L)	EOA (cm^2^)	AE (mJ)	Resistance (mmHg·min/L)	EOA (cm^2^)	AE (mJ)

FL 2.50A	0.508 ± 0.041	1.18 ± 0.020	74.6 ± 5.12	0.878 ± 0.065	1.14 ± 0.018	78.3 ± 5.67
FL 2.50B	0.389 ± 0.046*	1.23 ± 0.024*	64.6 ± 5.00*	1.14 ± 0.208*	1.14 ± 0.045	92.1 ± 4.14*

Change	0.119	0.05	10.0	0.262	0.00	13.8

* p < 0.05 versus FL 2.50A						

**B Retrograde intra-design variability as indicated by evaluation of duplicate prototypes from the FL 2.50 mm design**.						

	**STBR**					
		
Device	Regurgitation (mL)	Percent Reflux (%)	REL (mJ)	Energy Retention (%)		
		
FL 2.50A	11.0 ± 0.258	35.8 ± 1.63	9.55 ± 0.078	87.1 ± 1.18		
FL 2.50B	7.82 ± 1.17*	27.6 ± 5.10*	4.95 ± 1.44*	92.2 ± 2.77*		
Change	3.18	8.20	4.60	5.10		
	**STAF**					
FL 2.50A	2.19 ± 0.536	6.98 ± 1.71	0.400 ± 0.177	99.5 ± 0.227		
FL 2.50B	6.06 ± 1.30*	17.8 ± 3.58*	3.02 ± 1.21*	96.7 ± 1.20*		
		
Change	3.87	10.8	2.62	2.80		
		
* p < 0.05 versus FL 2.50A						

## Discussion

### a. Comparison with other studies

Several other published reports utilize similar hemodynamic conditions and parameters for evaluating prosthetic vein valves. More specifically, Delaria et al. [14] presented EOA data for fresh and fixed bioprosthetic valves with values around 1.1 and 0.70 cm^2^, respectively, at flow rates of 0.5 and 1 L/min. The fixed valves (that were suggested as most appropriate for native replacement) have smaller EOAs than the second-generation prototypes for which values of approximately 1.1 cm^2 ^were obtained for the SUAF simulation where the steady component flow rate was 0.75 L/min. However, for these bioprosthetic devices, EOA data was obtained by a visual method while it was calculated as a function of pressure and flow for our second generation valves. Delaria et al. also presented several cycles of a waveform that show reversing flow.

Pavcnik et al. [18-21] qualitatively described hemodynamic performance of their bioprosthetic valve. More specifically, "minimal or no leak" was noted after immediate venogram in twenty-four of twenty-five devices implanted in porcine inferior vena cavas. Of twenty-six valves implanted in ovines, twenty-five had "no reflux" on immediate venography and twenty-two upon sacrifice [18]. Two of the failed valves had "moderate reflux" at three months. In another ovine trial, four valves from a design array of forty-eight total valves had "significant leak" at six-week follow up on venogram [19]. Further, one of six orthogonally oriented valves "showed reflux" on venography compared to none of the six in anatomic orientation [20]. In patients, four of fifteen valves demonstrated "moderate leak" on venograms and ultrasound at twelve months [21].

With regard to prosthetic devices, Taheri et al. [22] reported regurgitant flow of 145 mL/min for a valve subjected to 300 mL/min of forward flow and back pressures of 100 mmHg. Further, *in vitro *test results of the prosthetic valve by Sathe et al. [23] showed that after more than 290,000 cycles the device had an opening pressure gradient of approximately 3 mmHg and a reflux rate of less than 3 mL/min at a pressure of over 150 mmHg. The valve was evaluated in an *in vitro *water system with a frequency of 40 cycles/min and an average proximal hydrostatic pressure of 65 mmHg. Unfortunately, the studies by Taheri et al. and Sathe et al. cannot be directly compared to each other or those of the second-generation prototypes. Hence, standardization of testing conditions and characterizing parameters are necessary as intended by our prior publication [26] and extended in the current manuscript.

### b. Device performance in 1:1 physiologically-based, *in vitro *flow system

The data of Figures 8 and 9 demonstrate that the *in vitro *flow system and corresponding parameters are capable of quantifying hemodynamic improvement from the CVI simulated "No Device" case relative to the second-generation prototypes. Also, the system is able to evaluate relative performance for device-specific selection as previously indicated by repeat measurements of the FL 1.25 mm prototype [26]. However, intra-design variability confounds design-specific selection, which is evident by the current study and also by comparing the data of the FL 1.25 mm prototype from our previous study [26] and another device of the same design that is subsequently presented. Therefore, the following selection process is device-, but not design-specific with regard to the second-generation prototypes.

### c. Relative importance of simulation position/condition and parameters

Devices were evaluated utilizing four physiologically relevant simulations by seven hemodynamic parameters without a single device being superior on all measures. Hence, selection must weigh the relative value of all parameters and simulations.

For the supine position simulations, Resistance and EOA were found to be significantly different from the "No Device" case for the four designs. However, all values are considered acceptable since they easily satisfied the acceptable performance criteria established *a priori *– namely, ΔP < 5 mmHg (≡ Resistance < 8.3 mmHg·min/L) and EOA > 80% of 'No Device' case (= 0.984 cm^2^), verifying that the devices did not notably impede antegrade flow.

With regard to the standing position simulations, STBR is the most demanding simulation for characterizing retrograde performance. This is because, in the STAF simulation, the higher frequency of the pumping cycle acts as an effective valve due to the reduced time available for the flow momentum to reverse. Hence, parameters of the STBR simulation are exclusively used in design selection. When considering specific parameters, most include reflux volume as an independent variable (with the exception of Antegrade Energy). As a result, there is inherent redundancy in the characterization of retrograde performance, which diminishes the multi-dimensional utility of the parameters in selection. Percent Reflux normalizes for periodic variations in antegrade volume and therefore, is reasoned as the only quantity necessary for selection of the second-generation prototypes.

While only one simulation and one parameter are necessary for device selection of the second-generation prototypes, a different combination of simulations and parameters may prove useful in distinguishing valves of more dissimilar designs. Of anticipated future importance are the Percent Reflux and Energy Retention parameters because they provide meaningful performance boundaries, which have physical significance. More specifically, Percent Reflux can be considered a valve ejection fraction where values greater than 100% are not possible and those less than 0% indicate a positive fluid accumulation in the distal venous space and CVI perpetuation. When the valve is operating in a range from 0 to 100% reflux, fluid accumulation in the distal venous space is negative and the conditions of CVI are being alleviated. Similarly, for the Energy Retention term, values less than 0% indicate the valve is allowing the hydrostatic fluid column to perform work on the calf muscle pump. Further, values greater than 0% indicate that the valve is permitting the energy exerted on the fluid from the calf muscle pump to be retained in the form of hydrostatic potential energy; hence, the term, Energy Retention. There is also an upper limit of 100% for the Energy Retention parameter. All second-generation prototypic, prosthetic valves perform in the optimal ranges of Percent Reflux and Energy Retention whereby the hemodynamics are favorable for CVI treatment.

### d. Selection

Based on their statistically superior performance as quantified by the Percent Reflux parameter in the STBR simulation, the most advanced second-generation valves featuring FLs of 2.50 and 3.75 mm are superior to those of 1.25 and 5.00 mm. However, as previously described, this selection is device- and not design-specific.

### e. Frame design

**Figure 10:**
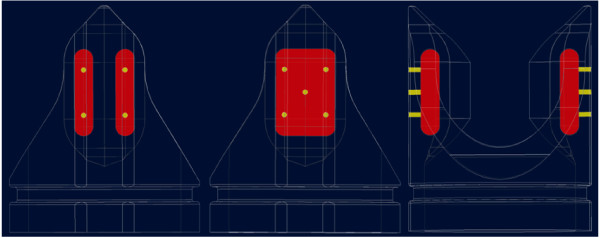
Potential drug delivery reservoir (red) and channel configurations (yellow) as part of the most advanced second-generation design.

A frame feature not evaluated via *in vitro *techniques and thus, not described in the Methods section is the incorporation of drug delivery reservoirs within each flange of the second-generation design. It is envisioned that the reservoirs communicate with the physiologic environment via channels made of a rate controlling material. The channels are directed either towards the valve lumen to influence thrombosis or towards the vessel wall to limit intimal hyperplasia and/or promote valve incorporation. Multiple isolated reservoirs within each flange allow a single device to contain more than one drug. In the single and dual reservoir per flange configurations, it is estimated that the reservoirs have volumes of 26.3 mm^3 ^and 7.5 mm^3^, respectively. Therefore, the single and dual reservoir configurations are estimated to have 56.6 mm^3 ^and 30.2 mm^3 ^of total volume per device, respectively. However, the approximations are based on the 3.75 mm FL design and vary directly with FL. Figure 10 depicts both the single and dual reservoirs with communicating channels. Also not evaluated by *in vitro *testing is a balloon catheter deliverable configuration whereby the frame base is composed of a radially expandable scaffold (similar to a stent) and the symmetric flanges are mated prior to deployment.

### f. Future directions

Prospective development objectives include: 1) the selection of an appropriate biomaterial for the frame, and, 2) casting BioSpan^® ^in a mold for more precise proximal edge contouring while scaling thickness for the 1× prototype (2.5 mil/0.0635 mm). It is also desirable to improve the reproducibility of leaflet-to-frame attachment to minimize intra-design variability. Finally, our group intends to report a CFD study of local hemodynamics including wall shear stress and residence time from the most advanced second-generation design prior to assessing biocompatibility *in vivo *utilizing an ovine external jugular vein model.

## Conclusion

Results indicate that the STBR simulation provided the best means of discriminating among second-generation valves of varying FLs. Further, only one parameter, Percent Reflux, was appropriate for device selection. Statistical analysis, revealed the 2.50 and 3.75 mm FL devices are superior to those of 1.25 and 5.00 mm with a Percent Reflux of 36%. However, intra-device and intra-design variability confound design selection. Nonetheless, all prototypic, prosthetic valves provided a dramatic and statistically significant improvement when compared to the "No Device" case (Percent Reflux of 205%), which was a simulated reference to the dynamic conditions of CVI. This leads to the general conclusion that the most advanced prototypic, prosthetic venous valve has the potential to reestablish normal antegrade circulation.

## Competing interests

The authors declare that they have no competing interests.

## Authors' contributions

Together, MTO and SER designed the second-generation venous valve with each individual contributing to certain features more than others. MTO produced the CAD models and constructed the mock-up and prototype valves from SLA frames and leaflet templates. MTO and SER both participated in data collection and together devised the new parameters with each individual contributing to certain terms more than others. MTO also analyzed data and drew conclusions.

## Appendix

Mathematically, the leaflet-closure plane point mapping was accomplished by first defining a variable, Θ_offset_, which is the angle formed by the x-axis, the origin, and the tangent point where the flange back meets the half circle. For the aforementioned optimized frame, Θ_offset _was found to be 12.7°. Then, the xy-coordinates of discreet points on the radial base edge were determined by incrementing the angle from the x-axis by 0.25 radians and applying the following equations:

(3) *X *= *R *cosΘ

(4) *Y *= *R *sinΘ

where X = the Cartesian coordinate of the radial base edge from the x-axis,

Y = the Cartesian coordinate of the radial base edge from the y-axis,

R = the valve radius, and

Θ = the angle from Θ_offset _in radians = angle – Θ_offset_.

Then, points on the radial base edge were mapped to their corresponding position on the flange using the following relationship:

(5) *S *= *R*Θ = *rθ*

where s = arc length,

R = the valve radius,

Θ = the angle from a point on the valve edge to Θ_offset _in radians,

r = the flange radius, and

θ = a reference angle corresponding to the mapped point on the flange.

In Equation 5, R was set at 6.25 mm and the solution of Equations 1 and 2 provided that r is 1.37 mm. Therefore, because Θ was incremented, θ is the dependent variable. Physically, θ is the angle formed by the θ_offset _reference point, the origin, and a specific point on the flange. Analogous to the radial base edge, two-dimensional Cartesian coordinates are found from the following equations:

(6) *x *= *r *cos *θ *+ *x*'

(7) *y *= *r *sin *θ *+ *y*'

where x = the Cartesian coordinate of the flange from the x-axis,

y = the Cartesian coordinate of the flange from the y-axis,

r = the flange radius,

θ = a reference angle corresponding to the mapped point on the flange,

x' = the x displacement of one flange circle, and

y' = the y displacement of the same flange circle.

The displacements are necessary because the points composing the flange are based on circles that have different centers. Thus, for the portion of the flange composed of the half-circle, x' equals (2r + Q) and for this specific design is 4.71 mm. Similarly, y' is r or 1.37 mm. For the quarter-circle portion, x' is Q or 1.97 mm and y' is r or 1.37 mm. The line connecting the tip of the flange and the origin is linear and therefore, requires a separate mathematical definition for mapping the remaining arc length of the radial base edge. These points were evenly distributed along the remainder of the closure path, which completed the two-dimensional considerations. Figure 4 depicts the Cartesian definitions for leaflet design in the transverse plane.

To resolve the coordinates in the third dimension, the frame was viewed in the yz-plane and the displacement in the z-direction was determined. For the radial base edge, the z-coordinate is zero by definition of the origin. Similarly, by design constraint, the z-coordinates of the closure plane all have a constant value. Staying consistent with nomenclature in the xy-plane, these relationships were expressed mathematically as:

(8) *Z *= 0

(9) *z *= *H*

where Z = the Cartesian coordinate of the radial base edge from the z-axis,

z = the Cartesian coordinate of the flange from the z-axis, and

H = the predetermined leaflet closure height.
